# A Case of Life‐Threatening Stridor: Vallecular Cyst in a 2‐Month‐Old Infant

**DOI:** 10.1002/ccr3.71797

**Published:** 2026-01-07

**Authors:** Abdur Rehman, Sibgha Fawad Memon, Muhammad Saad Anwaar, Anwar Ul Haq, Bilal Ahmad, Kamil Ahmad Kamil, Muhammad Kabir

**Affiliations:** ^1^ Saidu Medical College Swat Pakistan; ^2^ Peoples University of Medical and Health Sciences for Women Nawabshah Pakistan; ^3^ Federal Medical and Dental College Islamabad Pakistan; ^4^ Shifa International Hospital Islamabad Pakistan; ^5^ Internal Medicine Department Mirwais Regional Hospital Kandahar Afghanistan; ^6^ Department of Pediatric Surgery Saidu Group of Teaching Hospitals (SGTH) Swat Pakistan

**Keywords:** laryngoscopy, multidisciplinary intervention, respiratory distress, stridor in infants, surgical excision, vallecular cyst

## Abstract

Vallecular cysts are uncommon birth defects that substantially restrict an infant's airway. This paper emphasizes the difficulties in diagnosing and managing a transfer case with limited resources. If these cysts are not detected early, they can have major clinical consequences. A 2‐month‐old female infant presented with progressive inspiratory stridor, respiratory distress, vomiting, and fever over one month. Initial investigations suggested pneumonia, but direct laryngoscopy revealed a large vallecular cyst compressing the laryngeal structures. Blood cultures grew *Staphylococcus epidermidis* (MRSE). Emergency tracheostomy preceded complete cyst excision. Postoperative tracheal cultures identified XDR Acinetobacter, managed with targeted antibiotics.

**Key Clinical Message**

Infants with chronic stridor require immediate endoscopic assessment for vallecular cysts. It is a life‐threatening illness that requires prompt diagnosis and treatment to avoid complications. Multidisciplinary coordination is essential for airway stabilization and surgical management, particularly in complex cases with superimposed infections. Early excision prevents recurrence and mitigates life‐threatening complications.

## 1 Introduction

Tongue‐base cysts, also known as vallecular cysts, are an uncommon but potentially life‐threatening cause of stridor among young patients [1]. It can cause substantial upper airway obstruction, especially in neonates and babies. The vallecular cyst is a unilocular cystic mass, and its content is clear and noninfected fluid. These cysts, located in the vallecula between the base of the tongue and the epiglottis, are usually benign but can cause life‐threatening symptoms, including stridor, respiratory distress, feeding difficulties, and failure to thrive [2, 3, 4]. Vallecular cysts are thought to result from ductal blockage of mucous glands or embryological abnormalities [4].

Vallecular cysts typically appear within the first few weeks of life, with inspiratory stridor being a defining sign. If not addressed, they can lead to apnea, cyanosis, and even death [3, 5]. Vallecular cysts are hard to diagnose since they happen in about 3.49–5.3 out of every 100,000 neonates. There is a lot of overlap in symptoms with common respiratory infections, which might cause diagnostic delays. Flexible or direct laryngoscopy is the most effective method for diagnosing a lesion because it allows for dynamic visualization. Imaging modalities such as MRI or computed tomography (CT) scans can help determine the cyst's size and extent [4, 6]. Other congenital neck tumors that can be differentiated include thyroglossal duct cysts, dermoid cysts, and lymphangiomas [4].

To prevent airway blockage, vallecular cysts need to be surgically treated as soon as possible. Techniques such as marsupialization or complete excision are commonly used, with the latter being preferred due to lower recurrence rates [5, 6]. Early detection and treatment are crucial to avoiding problems and ensuring positive outcomes. This report describes a 2‐month‐old newborn with a vallecular cyst who presented with acute stridor and respiratory distress, highlighting the significance of multidisciplinary care in addressing this unusual but potentially lethal illness. This case underscores the vital role of expedited diagnosis and surgical intervention in preventing fatal airway compromise, particularly in complex transfer scenarios.

## 2 Case History, Examination, and Methods

A 2‐month‐old female infant was brought from Afghanistan for treatment of loud stridor and respiratory distress. She was in the usual state of health when she developed symptoms that began one month prior with progressive respiratory difficulty and vomiting. Her difficulty in breathing used to get worse over time. She had a previous ICU stay in Afghanistan for respiratory support and presented with a persistent low‐grade fever. Examination revealed tachypnea and loud inspiratory stridor, and she was oxygen dependent (about 2 L/min). An X‐ray of the chest revealed bilateral hilar and perihilar congestive changes and heterogeneous airspace opacification in the right lung upper zone. Laboratory findings included WBC 10,690/μL, hemoglobin 12.6 g/dL, and negative CRP. Blood cultures isolated methicillin‐resistant *Staphylococcus epidermidis* (MRSE), sensitive only to doxycycline and vancomycin.

**FIGURE 1:**
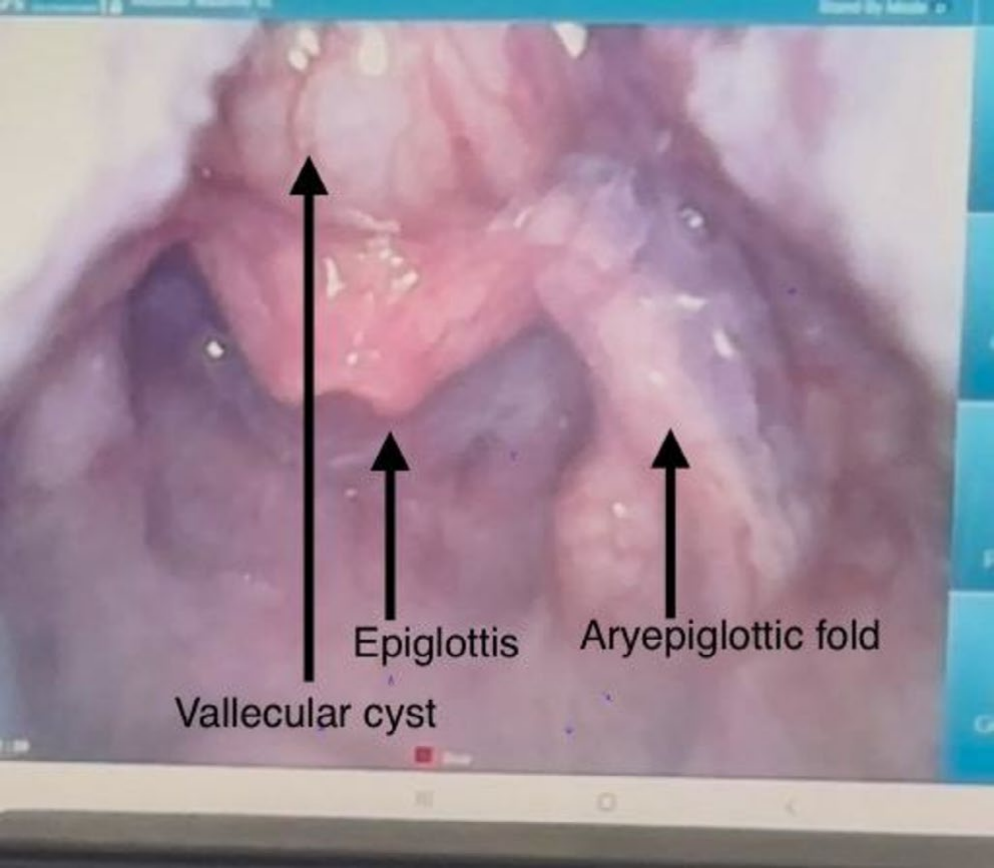
Vallecular cyst.

Bronchoscopy requested by the neonatologist led to direct laryngoscopy under anesthesia, which identified a large vallecular cyst between the tongue base and epiglottis compressing the aryepiglottic folds and arytenoids, as shown in Figure 1. Preoperative consultation was taken from the ENT surgeon, and a tracheostomy was planned prior to excision of the cyst. Tracheostomy was performed, and then excision of the cyst was done. The outer mucosal covering of the cyst was opened. The cyst was enucleated by dissecting it out from the surrounding structures. The cyst was completely excised; the cyst contained thick, mucoid, colorless secretions. The patient was transferred to the pediatric ICU for postoperative care. The patient was kept on low‐flow oxygen, 1.5 L, through tracheostomy. Oral feed was started, and subsequently, she was transferred to the room. Routine tracheostomy care was instituted by the respiratory therapist. Tracheal cultures later grew extensively drug‐resistant (XDR) Acinetobacter, sensitive to minocycline. The patient was discharged stable, with planned decannulation after resolution of the respiratory infection. Histopathology confirmed a benign vallecular cyst. At 6‐month follow‐up, she remained asymptomatic with no recurrence.

## 3 Discussion

(Congenital) vallecular cyst, an uncommon entity of laryngeal cyst, emerges from near or between the base of the tongue and the epiglottis. It is considered a benign retention cyst, characterized by the obstruction of submucosal glands [7].

Depending upon the size, it can be asymptomatic if small or present with respiratory distress, stridor, poor feeding, cyanosis, dysphagia, or airway obstruction, which can be fatal, especially in pediatric age groups [8, 9, 10]. In our case, the patient presented with fever, tachypnea, respiratory distress, and stridor, predominantly in the inspiratory phase, and several differential diagnoses were made by the attending physician, including upper respiratory tract infection (URTI), laryngomalacia, epiglottitis, laryngeal webs, and laryngeal cysts.

The overall incidence of vallecular cyst approximates between 3.49–5.3/100,000 newborns, as reported in an article published in 2023 [8]. Its anatomic location further makes it a challenging pathology, and several theories have been put forth for its mechanism of development that involve blockage of mucous glands and congenital deformity [11].

Various diagnostic modalities and imaging techniques are used to diagnose the cyst, including computed tomography (CT), flexible laryngoscopy, magnetic resonance imaging (MRI), direct laryngoscopy, and ultrasound [12, 13]. With further evolution in technology, prenatal diagnosis of vallecula is made possible nowadays with the help of ultrasound, usually done in the 2nd trimester of pregnancy, as reported in a study published in 2024 [2]. In our case, radiographic imaging and chest x‐rays were also performed because the symptoms were consistent with respiratory infection, and fever was one of the presenting complaints; moreover, her leukocyte count was raised, which was indicative of pathological invasion. To confirm the diagnosis, direct laryngoscopy, which is often used to rule out the diagnosis [13, 14], was done under general anesthesia, which revealed a cyst causing significant pressure on the arytenoid and aryepiglottic fold. Her blood culture was also sent, and staphylococcus infection was ruled out, confirming the concomitant presence of infection.

Another unique manifestation in this case was the need for tracheostomy, as the patient was in severe respiratory distress. A literature search suggests that immediate tracheostomy is mainly required when a patient's airway is compromised [15].

Treatment options for vallecular cysts comprise several options, mainly aiming to alleviate symptoms and prevent recurrence. This includes observation, aspiration and drainage, deroofing or marsupialization, laser or surgical excision, coblation and electrocautery (entcase) [13, 16, 17]. In this case, the cyst was first enucleated from surrounding structures, and then complete surgical excision was performed, which is the preferred method, as other modalities have a high recurrence rate [18].

## 4 Conclusion

This case exemplifies the critical importance of considering vallecular cysts in infants presenting with stridor unresponsive to conventional respiratory management. The diagnostic journey, international transfer, superimposed polymicrobial infections (MRSE and XDR Acinetobacter), and delayed identification underscores the need for rapid laryngoscopic evaluation in similar scenarios. Our experience confirms that complete surgical excision remains the definitive treatment to prevent recurrence, with tracheostomy serving as a vital bridge in severe airway compromise. The successful outcome hinged on multidisciplinary coordination across ENT, neonatology, and infectious disease teams. Future efforts should prioritize developing standardized protocols for early endoscopic assessment in resource‐limited settings and exploring minimally invasive techniques like coblation for high‐risk cases. Ultimately, heightened clinical suspicion of this rare entity can avert fatal complications in vulnerable infants.

## Author Contributions

**Abdur Rehman:** conceptualization, writing – original draft, writing – review and editing. **Sibgha Fawad Memon:** conceptualization, writing – original draft. **Muhammad Saad Anwaar:** data curation, writing – original draft. **Anwar Ul Haq:** investigation, project administration, supervision, writing – review and editing. **Bilal Ahmad:** writing – review and editing. **Kamil Ahmad Kamil:** conceptualization, writing – review and editing. **Muhammad Kabir:** supervision, writing – review and editing.

## Funding

The authors have nothing to report.

## Ethics Statement

We obtained written informed consent for the use of clinical data and images from the patient's guardian for publication of this case report. This study follows CARE guidelines for writing and reporting case reports. No Identifiable information is revealed and all patient data is anonymized to protect privacy.

## Conflicts of Interest

The authors declare no conflicts of interest.

## Data Availability

The data supporting the findings of this case report, including relevant clinical details and laboratory results, are available from the corresponding author on reasonable request. The patient consent form is also available upon reasonable request, following privacy and ethical guidelines.
